# Carbon reduction and corporate sustainability: Evidence from low-carbon city pilot policy

**DOI:** 10.1016/j.heliyon.2024.e28992

**Published:** 2024-04-02

**Authors:** Wenzhe Yu, Zhong Li, Caijuan Hu

**Affiliations:** a Zhejiang Gongshang University, Hangzhou, Zhejiang, China; b Zhejiang University of Finance and Economics Dongfang College, Haining, Zhejiang, China

**Keywords:** Low-carbon city pilot policy, ESG, Environment regulation, Crowding-out effect

## Abstract

The enhancement of corporate environmental, social, and governance (ESG) performance represents a crucial aspect of the broader green transformation agenda. Using a DID design, this paper examines the impact of China's low-carbon city pilot (LCCP) policy on the corporate ESG performance. Our findings demonstrate that the construction of LCCP exerts a positive influence on corporate ESG performance in pilot regions, particularly in industries and areas with high carbon emission intensity. Channel analyses reveal that LCCP policy heightens the environmental concerns of local governments and the public. Furthermore, LCCP policy has a crowding out effect with firms located in the surrounding cities. This paper responds to the calls for the determinants of ESG and enriches the understanding of policy impacts on corporate sustainability practices.

## Highlights

• The low-carbon city construction exerts a positive influence on corporate ESG performance in pilot regions.
• The relationship is heterogeneous across different carbon emission level.
• Low-carbon pilot policy has a crowding out effect on the ESG performance of firms in “peer” cities.
• Environmental concerns of the government and the public are the underlying channels.
• We provide comprehensive recommendations for policymakers to improve the ESG disclosure system.

## 1 Introduction

In response to the increasing carbon dioxide emissions, policymakers are implementing emission reduction governance measures. As primary economic actors in urban development, firms bear the responsibility of supporting urban sustainable development as a component of the urban low-carbon transition [1]. How firms can contribute to the low-carbon transformation of urban areas thus becomes a crucial research question [2]. At a specific country level, the State Council of China released *the Action Plan for Carbon Peaking before 2030* in October 2021, claiming that the industrial sector should lead the way in attaining carbon peaking by accelerating green, environmentally friendly and developing at a high level.

As the biggest producer of industrial products worldwide, China is up against several obstacles to reach "carbon neutrality" while carbon emissions are on rising. As one of its initiatives to cut carbon emissions at the local level, China has undertaken low-carbon city pilot (LCCP) construction in succession since 2010. According to the *Report on Climate Change 2021: Peak Carbon Neutrality Album*, which jointly compiled by authoritative experts, there were 18 cities with a score of 90 or above, or nearly 10% of the evaluated cities in 2020, compared to the early stage of LCCP in 2010. The findings suggest a substantial improvement in the extent of low-carbon development across the majority of Chinese cities. Moreover, LCCP initiatives demonstrate superior performance overall compared to non-LCCP efforts in fostering the green transition.

Recent research has emphasized the positive implications of constructing LCCP in terms of both the economy and society. On the one hand, such construction reduces carbon emissions by encouraging corporate technological innovation and boosting total factor productivity [3,4]. On the other hand, the LCCP policy mitigates urban pollution by modernizing the industrial structure, lowering energy consumption, and raising residents' green living standard [5,6]. However, a lack of studies has specifically addressed the LCCP policy may affect the corporate ESG performance from the micro viewpoint. Thus, this study aims to investigate corporate ESG performance under the influence of LCCP policies. Additionally, we aim to explore the potential crowding-out effect on surrounding cities.

To address these concerns and bridge the research gap, our focus is on examining the effectiveness and mechanisms of the LCCP policy on coporate ESG performance. The policy mandates each participating region to explore its unique low-carbon development mode and path in light of its economic conditions and available resources, as well as to formulate low-carbon development plans and greenhouse gas emissions reduction targets for evaluation purposes. This approach enables the accumulation of extensive low-carbon transformation experience through the LCCP policy [7].

In this paper, we adopt the second batch of LCCP as a setting, which is not affected by firms and regarded as a purely exogenous event for firms [8]. Employing a single-term DID approach, we find that the LCCP policy can improve corporate ESG performance, particularly in regions and industries with high carbon emission intensity. In addition, we also investigate the potential mechanisms of this study, featuring a key discussion on how the LCCP policy enhances environmental awareness among local governments and the public. Our results are robust to multiple dimensions of alternative measures of dependent variables, as well as various model specifications. Additionally, we show that the LCCP policy has a crowding-out effect on ESG performance of firms in nearby cities (peer firms).

This paper adds to the existing body of literature in four key aspects. Firstly, it enhances the understanding of micro-level impacts of the LCCP policy. Existing research mainly focuses on the impact of LCCP policy on technological innovation, total factor productivity and business risk [4]. Our paper is conducted from the view of corporate ESG performance, complementing this strand of literature [9,10]. Secondly, by exploring the intricate interplay of government environmental governance perspectives, this study deepens insights into the factors influencing corporate ESG performance. Existing research shows correlations between ESG performance in company characteristics, as well as, market attributes [[11], [12], [13]]. However, it's still unclear whether the bottom-up, all-encompassing environmental control regulations represented by the LCCP policy have an effect on corporate sustainability, as measured by ESG performance. Thirdly, this paper further discusses the crowding-out effects of the LCCP policy. While some prior research has predominantly concentrated on the impacts within the LCCP framework, this study expands the analysis to examine whether the policy implementation might lead to resource crowding-out in surrounding cities. By doing so, this research offers a more comprehensive understanding of the potential micro implications for both LCCP and non-LCCP. Fourth, the results provide practical guidance for policy-makers on how to adjust policy frameworks that can enhance corporate ESG performance and facilitate green transformation from a regional policy synergy perspective.

The remainder of the article is presented below: Section 2 conducts a comprehensive review of pertinent literature, outlines theoretical analysis and hypothesis development. Section 3 encompasses both the dataset and the model. Section 4 presents the empirical results. Section 5 delineates supplementary analyses, while Section 6 offering a concise summary.

## 2 Literature review, theoretical analysis and hypothesis development

### 2.1 The economic and environmental effects of LCCP

**Fig. 1:**
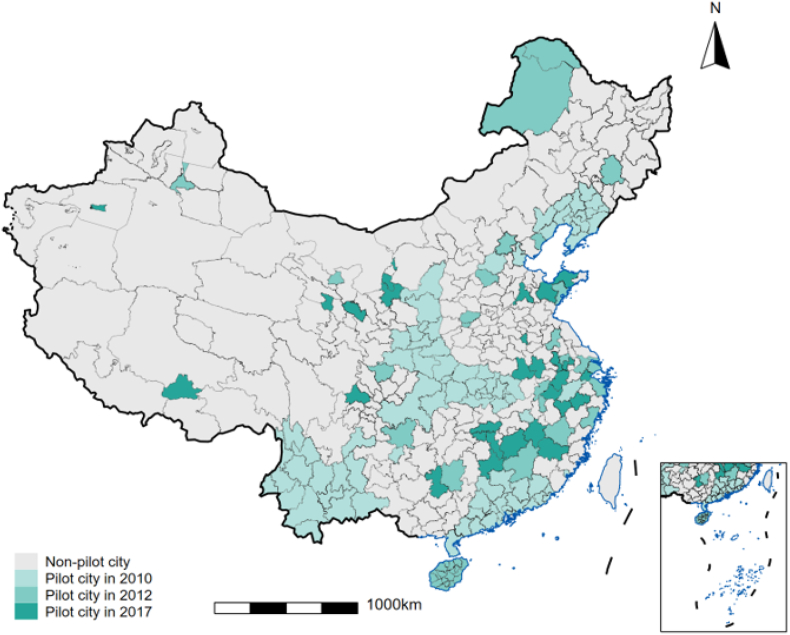
The map of the LCCP distribution.

In order to guarantee the targets that China set up in 2030 to limit greenhouse gas emissions, three batches of LCCP in 2010, 2012 and 2017 are established by the National Development and Reform Commission of China (See in Fig. 1). A number of previous studies have explored and delved into the potential consequences of the LCCP policy, mostly from the perspectives of economic gains and environmental effects.

From an economic perspective, the LCCP policy can assist pilot cities form economies of scale, so as to improve the overall green output, green total factor productivity and urban green innovations [9]. Specific to the micro firms subject, firms in LCCP has larger government subsidies, a larger reduction in taxes or a larger improvement in their financial position experienced a higher increase in R&D spending. With the increase of technological innovation and resource utilization efficiency, firms' total factor productivity will also increase [14]. In addition, firms located in LCCP have lower risks, such as systematic risk, etc. [15]. However, other scholars held a different view, arguing that LCCP may not have a positive effect and may actually hinder technological innovation capabilities. Firstly, LCCP policy increases the firms operating costs, thus indirectly affecting their investment in research and development [3]. Moreover, the policy incentives introduced by the government may increase firms’ reliance on government subsidies and the number of inefficient firms in the pilot cities, commonly referred to as "zombie firms" [16].

From the perspective of environmental impact, LCCP policy can affect carbon emission levels and efficiency by influencing energy and industrial structure [6]. However, some researchers held the opposite view, stating that LCCP policy leads to higher carbon intensity in the short term [17]. In addition to the analysis at the city level, it is also analyzed the mechanism from firms and residents' views. Chen et al.(2022) find that the elevation of local government officials will be influenced by environmental quality [18], which leading to the political promotion incentive effect makes sense. At the same time, LCCP policy reduces residents living carbon emissions through green living awareness and green habits, which has a guiding effect on residents' low-carbon choice behavior and is helpful to realize the carbon emission reduction plan in residents' life field [5].

### 2.2 Determinants of ESG performance

The current research predominantly focuses on examining the determinants of corporate ESG performance, encompassing factors at both the firm and market levels. In the realm of company characteristics, companies with family-controlled involvement, as well as gender and age differences among CEOs and board members,are the main factors [12,13,19]. In the context of market-level attributes, its influencing factors include national characteristics [20,21], regional social capital [22], political leaning [23] and cross-listed factors [24].

The topic of firms' economic behavior affected by environmental regulation has become discussed in recent academic research. The bulk of scholarly work exploring the link between ESG performance and environmental regulation has predominantly centered on the environmental protection dimension. Wang et al.(2022) discuss the important role of the central government's administrative means from the central environmental protection inspection [25]. Because of the different types of environmental regulations and property rights, there exist diverse impacts stemming from both market-driven and command-driven environmental policies [26].

### 2.3 Theoretical foundations of LCCP and ESG performance

Organizational legitimacy theory and signal theory function as frameworks for gaining insight into the motivations driving enterprises in pilot cities regarding ESG implementation within the context of LCCP policy.

Firstly, the theory of organizational legitimacy posits that legitimacy manifests in three principal forms: pragmatic, moral, and cognitive [27,28]. Central to this theory is the premise that legitimacy is a key antecedent resource. It not only directly impacts the continuity of enterprises but also plays a pivotal role in enabling them to secure other vital resources from the public domain, such as stakeholder support and governmental assistance [29,30]. The framework offers businesses a comprehensive lens through which they can understand and manage their societal legitimacy, thereby effectively shaping and preserving their reputation and garnering support from stakeholders, including governmental entities.

In the context of LCCP implementation, local governments have introduced more stringent punitive measures for enterprises that fail to comply with environmental standards, with a particular emphasis on those exhibiting high carbon emissions. This heightened regulatory scrutiny underscores the importance of sustainable development at the local level. As a result, enterprises in pilot cities are experiencing increased pressure to maintain their legitimacy, compelling them to adopt ESG practices [16]. This dynamic illustrates the intersection of organizational legitimacy and environmental policy, highlighting the influence of regulatory frameworks on corporate behavior.

Then, the signal theory, rooted in the dynamics of market interactions under conditions of asymmetric information between buyers and sellers, addresses the challenges of information asymmetry in competitive settings [31]. Companies bridge informational gaps by disseminating pertinent information to various stakeholders, including the market, investors, and the broader public. This strategic communication mitigates potential conflicts and garners enhanced external support [32,33]. In the context of the LCCP policy, there is an increased focus from both government and public on corporate environmental practices. Within this framework, management figures such as executives, directors, and managers play a pivotal role as signalers. They leverage ESG criteria as a signaling tool, directing information flows to signal receivers like investors and regulatory bodies. This approach aligns more closely with market demands, thereby ameliorating issues of information asymmetry [34].

### 2.4 Hypothesis development

Due to the presence of non-economic activities with externalities, ecological environmental governance requires financial backing from firms [35]. However, it reduces production efficiency and firms are thus reluctant to take the initiative in environmental governance [36]. The LCCP policy is an obligatory environmental regulation for local governments as well as companies, which is a comprehensive "bottom-up" approach in which the central government provides overall national targets and LCCP sets their own low-carbon targets and innovative energy efficiency schemes. The LCCP policy can have effects on company ESG performance in following aspects.

First, LCCP policy can help create low-carbon entry barriers, increase the cost of non-compliance, enhance firms' motivation to improve environmental governance, and establish a policy forcing mechanism [18]. Local governments establish carbon emission standards and formulate corresponding supervision and punishment measures according to the production and development characteristics of different industries, while good corporate ESG performance can guarantee their "legitimacy" and environmental reputation [30].

Moreover, from the perspective of policy incentives, local government officials in LCCP are motivated to build a low-carbon ecosystem through multi-subject interaction for the purpose of "political competition", as well as, provide a multi-dimensional support as much as possible and increase government investment in environmental protection [16]. In this manner, it will fully leverage the incentive effect of LCCP. Firms that make an effective effort to practice their ESG engagement are aligned with the aims of local governments' low-carbon transformation in the face of global systemic threats caused by climate change. This, in turn, is conducive to obtaining more policy support and improving market advantages.

Furthermore, from the perspective of policy promotion, in LCCP, local governments strengthen the publicity of low-carbon environmental protection, and the public's awareness of energy conservation and emission reduction will largely increases [5]. Consumers and investors tend to be more optimistic about firms with better ESG performance [37]. The market-based mechanism encourages firms to improve ESG performance, so as to transmitting a good "green signal" to the stakeholders [38].

H1 Treatment firms experience an improvement in ESG performance after the adoption of LCCP policy.

In addition to having an impact in the pilot jurisdiction, the "first mover" label of the LCCP will put enormous institutional pressure on the "peers" (non-LCCP), producing crowding out effect. The horizontal competition of government competition theory explains the reasons [39]: adjacent "peers" often exhibit homogeneity, and competition between jurisdictions results in a reduction in the acquisition of "peers" green capital and a decrease in green financial subsidies [40,41]. This ultimately leads to insufficient supply of public goods resources, such as environmental investments, thereby affecting the ESG of "peer" companies.

Firstly, it rises the difficulty of obtaining high-quality environmental resources. To some extent, in terms of resource acquisition in environmental governance, the unapproved cities are in a disadvantageous position. The strategy of "upward competition" of local governments leads to increasing pressure of environmental resource competition of government agencies [42]. Secondly, it increases the cost of acquiring economic resources. For non-LCCP, low environmental legitimacy will make it subject to extra environmental supervision. For example, stakeholders require firms to deliver more environmental improvement signals, leading to higher pollution control costs and financing pressure for firms [43], as well as, leading to the more difficulty for firms in non-LCCP to practice ESG. Finally, it weakens the political resources of local officials. In the process of official selection and promotion [44], Chinese government often has a political championship. Specifically, the three batches of LCCP provides a benchmark for other cities to imitate. "Peer" local officials will seize the opportunity and allocate more political resources to pilot areas, so as to improve their "green achievements" during their term of office and crowd out political resources in non-pilot areas.

Based on theories of government horizontal competition, the following hypotheses are presented.

H2 LCCP policy will have crowding-out effects on the ESG performance of "peer" firms (non-pilot areas in the same province).

**Fig. 2:**
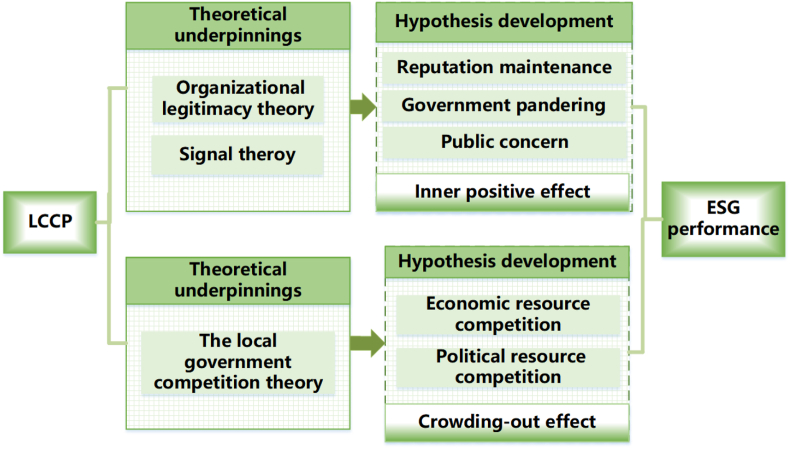
Theoretical framework.3. Research Design.

Fig. 2 presents the theoretical framework, tracing from its inception to hypothesis development, specifically including its positive effects and potential crowding-out effects.

### 2.5 Data and sample

To assess the main hypothesis, we utilize data from Chinese A-share listed firms during the sampling period spanning from 2010 to 2016. The chosen sample time frame aims to exclude interference from both internal and external policies. Firstly, we select the sample interval starting from the year 2010 to reduce the interference of the first batch of LCCP on the select"ed sample. As the implementation intensity of the first batch of LCCP may have been limited in the early stage, and the intervention time of the two batches of LCCP is narrow, we take 2010 as the starting year of the sample. Secondly, the sample ending in 2016 aims to reduce interference from other policies. For example, in 2017, Chinese seven departments jointly issued the Green Finance Innovation Reform Pilot Zone Overall Plan in 5 provinces and 8 cities. Such policies may affect firms' behavior regarding environmental responsibility. In order to mitigate the influence of concurrent policies, we choose 2016 as the endpoint for our sample period. During the experimental pilot selection process, we initially exclude firms from the first batch of LCCP to avoid interaction and designate the second batch as the treatment group, while the remaining firms comprised the control group. We apply specific criteria, including the exclusion of the finance and insurance sectors, special treatment, and firms lacking essential variable information. This process yield a final dataset of 10,239 observations. To address potential biases from outliers, all continuous variables were winsorized at the 1% and 99% levels.

### 2.6 Empirical model and variable definitions

According to the institutional background presented in previous, the LCCP policy provides an ideal setting for investigating the real impact of this environmental constraints, by employing DID approach to examine the influence on corporation ESG performance. We take the second batch of LCCP as the "treatment group". To avoid interference from the first batch of LCCP and get the "net effect", we define the samples after deleting the first batch of LCCP as the "control group". The model is conducted with city clustering adjusted standard error, which is shown as follows:

(1) $${ESG}_{i,t+1}=\alpha_{0}+\alpha_{1}{Treat}_{i}*{Post}_{t}+\alpha_{2}{Controls}_{i,t}+Firm\;FE+Year\;FE+City\;FE+\varepsilon_{i,t}$$

Where i represents a firm, t indicates a year. ESG is an indicator to assess corporate ESG performance. In alignment with Zhai et al.(2022), we adopt Huazheng ESG rating, provided by Sino-Securities Index Information Service (Shanghai) Co. Ltd., to assess corporate ESG performance. This index categorizes companies into nine tiers: AAA, AA, A, BBB, BB, B, CCC, CC, and C, facilitating a comprehensive evaluation of their ESG practices [45]. We assign them as "9-1" respectively, and the "9" indicates the highest level of firms' ESG engagement corresponding to original rating "AAA". As detailed in Table 1, both ${Post}_{t}$ and ${Treat}_{i}$ are dummy variables. Our focus centers on ***β***1, the estimated coefficient of ${Treat}_{i}*{Post}_{t}$, which implies the impacts of LCCP policy on corporate ESG performance. Following prior studies on the determinants of ESG performance [22,47,48], we also induce a number of control variables, the definitions of which are outlined for further clarity in Table 1.

**Table 1:** Variable definitions.

Variables	Definitions
${ESG}_{t+1}$	The Huazheng ESG rating index of lags one stage.
*Treat*	If firms registered in the second batch of LCCP, *Treat* = 1,and 0 else.
*Post*	*Post* = 0 for 2010–2012, and *Post* = 1 for 2013–2016^1^.
*Size*	Taking the natural logarithm of book assets.
*Lev*	Dividing total liabilities by total assets.
*ROA*	Dividing net profit by average total assets.
*CF*	Dividing operating cash flow by total assets.
*Growth*	The differences of firm's operating income for the current and the previous year divided by the operating income.
*Indep*	The proportion of independent directors to the total number of board members.
*Board*	The natural logarithm of the total count of board members.
*Dual*	A dummy variable is defined as 1 if the chairman are the same individual with manager (*Dual* = 1), and 0 else.
*Top1*	The fraction of shares held by the largest shareholder.
*SOE*	*SOE* = 1 for the firms is state-owned, and 0 else.

## 3 Empirical results

### 3.1 Descriptive statistics

The primary variables' descriptions are presented in Table 2. Out of the 10,239 observations, the average ESG rating, lagged by one stage, stands at 6.612. This suggests significant variability in ESG disclosure scores across the firms sampled. Additionally, the mean values for the main binary variables of *Post* and *Treat* are 0.642,0.375. This shows the ratio of sample after the establishment of LCCP account for 64.2%, and the treatment group accounts for 37.5%. In addition, we can also see the averages of city CO_2_ emissions and industrial CO_2_ as 71.253 million tons and 322.438 million tons, respectively. This suggests significant variation in CO2 emissions among the sample firms^2^. The main variables are in line with prior literature in Table 2 [4,[49], [50], [51], [52], [53]].

**Table 2:** Descriptive statistics.

*Variables*	N	Mean	SD	Min	P25	P50	P75	Max
${ESG}_{t+1}$	10,239	6.612	1.076	4.000	6.000	6.000	7.000	9.000
*Treat*	10,239	0.375	0.484	0.000	0.000	0.000	1.000	1.000
*Post*	10,239	0.642	0.479	0.000	0.000	1.000	1.000	1.000
Treat*Post	10,239	0.242	0.428	0.000	0.000	0.000	0.000	1.000
*Size*	10,239	22.07	1.286	19.720	21.140	21.880	22.780	26.100
*Lev*	10,239	0.435	0.213	0.051	0.262	0.428	0.604	0.894
*ROA*	10,239	0.038	0.051	−0.164	0.013	0.035	0.064	0.186
*CF*	10,239	0.042	0.071	−0.168	0.003	0.042	0.085	0.239
*Growth*	10,239	0.183	0.453	−0.540	−0.032	0.106	0.276	3.004
*Indep*	10,239	0.38	0.07	0.250	0.333	0.364	0.429	0.600
*Board*	10,239	2.298	0.243	1.609	2.197	2.303	2.485	2.890
*Dual*	10,239	0.227	0.419	0.000	0.000	0.000	0.000	1.000
*Top1*	10,239	35.67	15.28	8.450	23.570	33.680	46.050	75.920
*SOE*	10,239	0.423	0.494	0.000	0.000	0.000	1.000	1.000
*City_CO*_*2*_	9,533	71.253	57.703	1.137	35.513	61.783	78.244	230.712
*Industy_CO*_*2*_	6,917	322.438	706.806	0.104	14.063	28.156	305.274	3669.338

### 3.2 Main results

Table 3 presents the foundational findings, indicating that even after adding control variables, the coefficients associated with Treat*Post remain consistently positive and statistically significant at the 1% level. Specifically, the coefficients are 0.085 with a corresponding t-value of 2.66, and 0.085 with a t-value of 2.71, respectively. It implies that there is a notable enhancement in corporate ESG performance within the LCCP framework, thereby providing support for hypothesis 1.

**Table 3:** Baseline tests.

*Variable*s	(1)	(2)
	${ESG}_{t+1}$	${ESG}_{t+1}$
Treat*Post	0.085***	0.085***
	**(2.66)**	**(2.71)**
*Size*		0.159***
		(5.41)
*Lev*		−0.456***
		(-4.33)
*ROA*		0.594**
		(2.14)
*CF*		0.062
		(0.61)
*Growth*		−0.028*
		(-1.76)
*Indep*		−0.119
		(-1.04)
*Board*		−0.056
		(-1.30)
*Dual*		−0.027
		(-1.00)
*Top1*		0.002
		(0.89)
*SOE*		0.138
		(1.59)
*Constant*	6.516***	3.286***
	(290.79)	(5.29)
Year FE	YES	YES
Firm FE	YES	YES
City FE	YES	YES
Observations	10,239	10,239
Adj R^2^	0.683	0.687

**Note:** The t-statistics in parentheses are standard errors. Significance levels are denoted by ***, **, and *, indicating significance at the 1%, 5%, and 10% levels, respectively.

### 3.3 Robustness test

#### 3.3.1 Parallel trend assumption

**Fig. 3:**
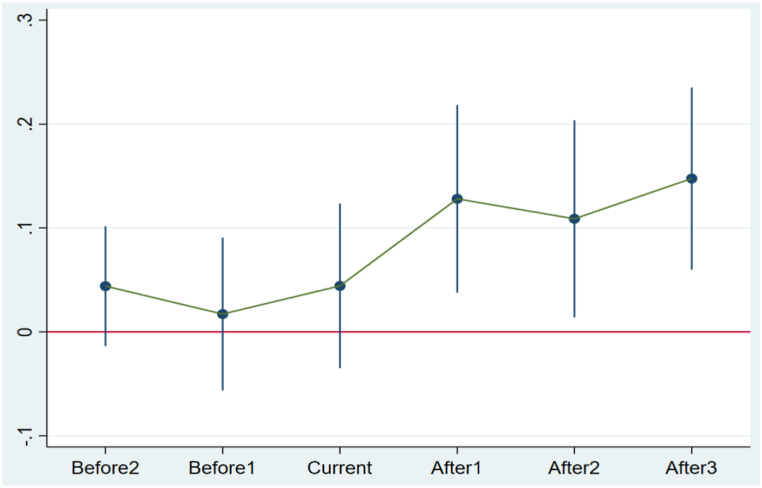
Parallel trend diagram of the empirical model.

The DID model is built upon a crucial presupposition: that there exist no discrepancies between the treatment and control groups before the policy implementation takes effect. The results show that before the implementation of the LCCP policy, the coefficients of interaction (Treat*Before2, Treat*Before1, Treat*Current) are not significant; however, following the enactment of the LCCP policy, the coefficients of the interaction terms become significant (Treat*After1, Treat*After2, Treat*After3), thus fulfilling the parallel trend assumption (See Table 4 and Fig. 3).

**Table 4:** Parallel trend assumption.

*Variables*	${ESG}_{t+1}$
Treat*Before2	0.044
	(1.26)
Treat*Before1	0.017
	(0.39)
Treat*Current	0.044
	(0.92)
Treat*After1	0.128**
	**(2.34)**
Treat*After2	0.109*
	**(1.89)**
Treat*After3	0.148***
	**(2.78)**
*Controls*	YES
Year FE	YES
Firm FE	YES
City FE	YES
Observations	10,239
Adj R^2^	0.687

**Note:** The table displays the outcomes of verifying the parallel trend assumptions within the DID model. T-statistics enclosed in parentheses are computed using standard errors. Significance levels are denoted by ***, **, and *, indicating significance at the 1%, 5%, and 10% thresholds, respectively.

#### 3.3.2 Endogeneity

(1) PSM-DID

The above multiple regression analysis in the benchmark model may suffer from the endogeneity issue. Following previous studies [14] and considering the p value and economic significance, we employ the propensity score matching (PSM) technique to mitigate sample selection bias. Specifically, the matching variables are consistent with the baseline model's control variables, and we match the firms registered in LCCP (the treatment group) with the firms which are not registered in LCCP (the control group) using nearest-neighbor matching with ratios of 1:1, 1:2, and 1:3, respectively, based on a range of ±10% around the propensity score. The results align with the baseline when controlling for firm, year and city level, demonstrating that the fundamental findings remain unaltered even when factors that were not observed are left out (see in Table 5).

(2) SYS-GMM

**Table 5:** PSM-DID.

*Variable*s	PSM-DID					
	1:1	1:1	1:2	1:2	1:4	1:4
Treat*Post	0.076**	0.075**	0.170**	0.144*	0.208**	0.223**
	**(2.07)**	**(2.11)**	**(2.02)**	**(1.76)**	**(2.32)**	**(2.60)**
*Controls*	NO	YES	NO	YES	NO	YES
Year FE	YES	YES	YES	YES	YES	YES
Firm FE	YES	YES	YES	YES	YES	YES
City FE	YES	YES	YES	YES	YES	YES
Observations	6,965	6,965	4,275	4,275	3,909	3,909
Adj R^2^	0.681	0.687	0.671	0.678	0.677	0.684

**Note:** The annotation in this table remains consistent with that in Table 3.

To address endogeneity issues and mitigate self-selection bias, we employ the system GMM approach [[54], [55], [56]]. It is constructed as follows:

(2) $${ESG}_{i,t+1}=\alpha_{0}+\alpha_{1}{ESG}_{i,t}+\beta_{1}{{Treat}_{i}*Post}_{t}+\gamma_{1}Controls+Firm\;FE+Year\;FE+City\;FE+\varepsilon_{i,t}$$

As presented in Table 6, we show the FE and SYS-GMM estimation to the model. The p values of the AR (1) is within the significance level of 0.1, and the AR (2) test exceed the significance level, indicating the existence of first-order sequence auto-correlation and absence of second-order sequence auto-correlation in the regression process of this section of the model and thereby overcoming the endogeneity problem. Moreover, the Hansen J-test value indicates no over-identification.

**Table 6:** The comparison bettewn FE and System GMM estimation.

*Variables*	FE estimation	SYS-GMM estimation
Treat*Post	0.052**	0.097***
	**(2.39)**	**(3.03)**
*Controls*	YES	YES
Year FE	YES	YES
Firm FE	YES	YES
City FE	YES	YES
AR(1)	/	[0.000]
AR(2)	/	[0.292]
Hansen Test	/	[0.104]
Observations	10,239	10,239
Adj R^2^	0.716	/

**Note:** The brackets corresponding to AR(1), AR(2), and the Hansen Test represent the P-values for the first-order auto-correlation, second-order auto-correlation tests, and the Hansen over-identification test, respectively.

#### 3.3.3 Alternative measure of ESG

Because there is a high variability of ESG scores and the "noise" generated by different measurement methods may potentially impact the research findings [57], we shift to three categories alternative measures of ESG. Firstly, we replace the ESG rating data with those from other rating agencies. Following He et al. (2023), the data are retrieved from Bloomberg database and RKS. The higher *ESGbloom* and *RKS*, the better ESG performance [58]. Secondly, we re-measure the ESG rating based on the Sino-Securities Index ESG Ratings. To avoid interference from arbitrary classifications, this paper replaces the measurement of ESG performance to 1–3 ranks. We assign 1 to "C-CCC", 2 to "B-BBB", and 3 to "A-AAA", resulting in *ESG_1*. Additionally, *ESG_dum* = 1 if the ESG rating is "A-AAA", and 0 otherwise [59]. Thirdly, we comprehensive consider ESG indicator variables. Specifically, we combine Huazheng with Bloomberg ESG rating index (*Mean_1*) and merge Huazheng, Bloomberg, RKS CSR ratings together (*Mean_2*). In Table 7, the core coefficient is 0.753, 1.564, 0.093, 0.031, 0.432, 0.462, respectively (with the t value is 1.86, 2.05, 0.093, 0.031, 0.432, 0.492). It come to the same conclusions that LCCP policy can positively improve the corporate ESG performance.

**Table 7:** Alternative measure of ESG.

*Variables*	Substitution		Measurement change		Average	
	${ESGbloom}_{t+1}$	*RKS*_*t+1*_	*ESG_dum*_*t+1*_	*ESG_1*_*t+1*_	${Mean\_1}_{t+1}$	${Mean\_2}_{t+1}$
Treat*Post	0.753*	1.564**	0.093*	0.031*	0.432**	0.492**
	**(1.86)**	**(2.05)**	**(1.81)**	**(1.81)**	**(2.03)**	**(2.12)**
*Controls*	YES	YES	YES	YES	YES	YES
Year FE	YES	YES	YES	YES	YES	YES
Firm FE	YES	YES	YES	YES	YES	YES
City FE	YES	YES	YES	YES	YES	YES
Observations	3,991	2,732	10,139	10,139	3,210	1,992
Adj R^2^	0.803	0.843	0.659	0.659	0.820	0.899

**Note:** The annotation in this table remains consistent with that in Table 3.

#### 3.3.4 Addition of new control variables

The characteristics of company executives can shape corporate decisions. For example, female board members might demonstrate higher effectiveness in strategic decision-making when compared to their male counterparts [60,61]. Therefore, we further consider other corporate governance control variables, including the gender diversity of the board [62,63], CEO's age [64]. Specifically, we use female directors percentage within the board of directors to reflect the gender diversity (*BCG*), as well as, define the average age of the CEO in that year to evaluate the age of managers (*CEO_meanAge*).

Moreover, factors external to the company, such as the operational environment and country of origin, can also be considered in control variables [20,21]. Similarly, since firms are economic agents of city development, we have reasons to believe that the level of cities development may affect the corporate ESG performance. Thus, we control GD per capita, measured by the logarithm value of GDP per capita to mitigate the concern that city-level characteristics could affect corporate ESG performance [23,65].

As shown in Table 8, our analysis demonstrates that upon integrating city-level variables and newly introduced corporate governance control variables, the main result is still robust, namely, the LCCP policy improves the corporate ESG performance.

**Table 8:** Robustness test with adding new control variables.

*Variables*	(1)	(2)	(3)	(4)
	${ESG}_{t+1}$	${ESG}_{t+1}$	${ESG}_{t+1}$	${ESG}_{t+1}$
Treat*Post	0.085***	0.103***	0.082**	0.102**
	**(2.71)**	**(2.68)**	**(2.45)**	**(2.56)**
*Original Control Variables (firm-level)*	YES	YES	YES	YES
*New Control Variables (city-level)*	NO	YES	NO	YES
*New Control Variables (BGD)*	NO	NO	YES	YES
*New Control Variables (CEO characteristic)*	NO	NO	YES	YES
Year FE	YES	YES	YES	YES
Firm FE	YES	YES	YES	YES
City FE	YES	YES	YES	YES
Observations	10,239	9,492	9,283	9,272
Adj R^2^	0.687	0.691	0.692	0.692

**Note:** The annotation in this table remains consistent with that in Table 3.

#### 3.3.5 Staggered DID

As mentioned above, LCCP has so far been established in three batches. The baseline model applies the standard DID, which excluding the samples of first and third batches of LCCP, thus obtaining unbiased coefficient estimates. Although staggered DID may result in bias [66], it can help us approximately examine whether the core conclusions apply to all three batches of LCCP. This paper imitates the existing literature [67], and extend the sample period to 2007–2021 to robust test the main findings. The main explanatory factor in this model is the dummy variable *DID*. *DID* = 1 in that year and any succeeding years if a city is designated as a LCCP, otherwise 0. The results still demonstrate the promotion effect of LCCP policy (see in Table 9).

**Table 9:** Robustness test with staggered DID estimation.

Variables	(1)	(2)
	${ESG}_{t+1}$	${ESG}_{t+1}$
***DID***	0.094**	0.096**
	**(2.02)**	**(2.12)**
*Controls*	NO	YES
Year FE	YES	YES
Firm FE	YES	YES
City FE	YES	YES
Observations	29,160	29,160
Adj R^2^	0.541	0.567

**Note:** The annotation in this table remains consistent with that in Table 3.

## 4 Further analyses

### 4.1 Heterogeneity analyses

#### 4.1.1 The heterogeneous effects of carbon emissions

As a comprehensive means of environmental regulation, LCCP policy requires local governments in pilot areas to adjust industrial layout, optimize energy structure, reduce energy consumption etc., creating pressure for firms' carbon emissions. In general, the LCCP policy has certain flexibility [6]. On the one hand, each LCCP can explore its own mode of low-carbon development and devise sustainable development strategies tailored to its distinct economic status and resource endowment. Therefore, local governments will make efforts to manage firms' carbon emission reduction based on the differences in carbon emission levels among cities. On the other hand, taking account of the industry-specific characteristics, local governments will introduce corresponding industrial policies, fiscal policies and tax policies. Specifically, firms in high-carbon industries face greater emission reduction costs. Strict emission standards will force high-carbon industries to carry out green transformation and guide high-carbon firms to fulfill their social responsibilities more actively.

Panel A report the heterogeneous effects of city-level carbon emission. In Columns (3) and (4), we include the *City_CO*_*2*_ indicator^3^: cities with high carbon intensity and cities with low carbon intensity (*City_CO*_*2*_ = 1 and *City_CO*_*2*_ = 0, respectively). The results show that, for firms registered in cities with higher carbon intensity, the coefficient of Treat*Post is 0.099 (*t* = 1.80). However, the core coefficient is not statistically significant in Columns (2).

Columns (3) and (4) demonstrate the heterogeneous effects of industry-level carbon emission. Specifically, we take the median industrial carbon emission intensity as the cut-off point: high-carbon industry and low-carbon industry (*Industy_CO*_*2*_). Column (4) shows that, for the subsample with high industrial carbon dioxide emission intensity (*Industy_CO*_*2*_ = 1), the core coefficient is positive (t = 2.03). In contrast, for the low industrial carbon dioxide emission intensity group (*Industy_CO*_*2*_ = 0), the core coefficient is not statistically significant, which implies that the LCCP policy has different impacts on the ESG performance of industries with different carbon emission intensity.

#### 4.1.2 The heterogeneous effects of firm-level characteristic

Besides, firms' individual characteristic may lead to heterogeneity of ESG performance. Firstly, under the circumstances of "Chinese-style decentralization", fiscal decentralization and promotion incentive have an important impact on the local governments' decision-making. In contrast to non-state-owned firms, the primary advantage of Chinese state-owned firms lies in their access to political resources provided by the government [68]. Without an effective mechanism to separate business from government influence [69,70], state-owned firms may serve as a breeding ground for improved political performance. Therefore, local governments in LCCP give financial support and preferential policies provided by the central government priority to state-owned firms, promoting them to be more motivated to participate in environmental and social issues. Secondly, constraints on financing, representing a particular aspect of a company's economic resources, can significantly impact its capital investments and profitability [71,72]. In the process of promoting LCCP policy, firms with higher financing constraints will have more motivation and pressure to improve their green image, so as to striving for more economic resource and maximize benefits.

In Panel B, Columns (1) and (2) demonstrate the diverse impacts of property rights. The dataset is devided into two groups: privately owned firms (*SOE = 0*) and state-owned firms (*SOE = 1*). For state-owned firms, the coefficient of Treat*Post is 0.098 (t = 2.40). In contrast, for privately owned firms, the primary explanatory variable fails to attain statistical significance.

The heterogeneous effects of financial constraints can be seen in Panel B. Following Kaplan and Zingales(1997), we use financial constraints of *KZ* index [73]. The core coefficient in the group of firms with high degree of *KZ* index is 0.109 (t = 2.25). However, in the group of firms with low degree of *KZ* index, the Treat*Post is not statistically significant.

#### 4.1.3 The heterogeneous effects of greenwashing risk

In recent years, many scholars have confirmed that the company's greenwashing behavior and ESG performance are closely related. A high variability of ESG scores will lead to the probability of greenwashing [74]. While green risk and ESG performance are not a mere reverse variation relationship [75], green risk changes corporate decision-making in response to environmental regulatory policies. According to the theory of signals and resource competition, when faced with the pressure of LCCP policy, ESG disagreement, which lead to greenwashing, has a stronger incentive to improve the pilot cities' ESG performance, thereby sending positive signals to markets and stakeholders to improve the availability of resources competition.

Therefore, we further discuss whether corporate greenwashing risk affects the LCCP policy in promoting the ESG performance. Following Zhang(2023), we adopt the approach to measurement of corporate greenwashing, which defines it as the difference between standardized ESG scores and ESG performance scores [75]. In details, the variable ${greenwash}_{1}$ is adopted by Bloomberg ESG to measure standardized ESG disclosure, whereas Huazheng ESG focuses on ESG performance, The variable ${greenwash}_{2}$ is used Bloomberg ESG data for measuring ESG disclosure, while the news rating index RKS CSR rating for the ESG performance.

Panel C show the heterogeneous effects of company greenwashing. greenwash is a dummy variables, which means the high degree of greenwashing (greenwash = 1) and the low degree of greenwashing (greenwash = 0), respectively. Table 10 illustrates that the LCCP policy significantly promotes corporate practices associated with higher greenwashing risk. It implies that the positive effects of LCCP are more apparent when enterprises face larger differences in ESG ratings, and an increase in the number of external rating agencies contributes to reducing information asymmetry and improving the performance of enterprises' environmental responsibility [76].

**Table 10:** Cross-sectional analyses.

Panel A: carbon emissions				
*Variable*s	(1)	(2)	(3)	(4)
	City_CO_2_ = 0	City_CO_2_ = 1	Industy_CO_2_ = 0	Industy_CO_2_ = 1
Treat*Post	**−0.018**	0.099*	**0.092**	0.135**
	**(-0.21)**	**(1.80)**	**(1.39)**	**(2.03)**
*Controls*	YES	YES	YES	YES
Year FE	YES	YES	YES	YES
Firm FE	YES	YES	YES	YES
City FE	YES	YES	YES	YES
Observations	4,726	4,807	3,398	3,519
Adj R^2^	0.673	0.705	0.710	0.632

Panel B: company characteristic				
*Variable*s	(1)	(2)	(3)	(4)
	SOE = 0	SOE = 1	KZ = 0	KZ = 1
Treat*Post	**0.059**	0.098**	**−0.008**	0.109**
	**(1.36)**	**(2.40)**	**(-0.15)**	**(2.25)**
*Controls*	YES	YES	YES	YES
Year FE	YES	YES	YES	YES
Firm FE	YES	YES	YES	YES
City FE	YES	YES	YES	YES
Observations	5,907	4,332	4,402	4,402
Adj R^2^	0.638	0.707	0.706	0.698

Panel C: company greenwashing				
*Variable*s	(1)	(2)	(3)	(4)
	${\text{greenwash}}_{1}$ = 0	${\text{greenwash}}_{1}$ = 1	${\text{greenwash}}_{2}$ = 0	${\text{greenwash}}_{2}$ = 1
Treat*Post	**0.076**	0.252***	**0.008**	0.162**
	**(0.83)**	**(2.65)**	**(0.08)**	**(2.01)**
*Controls*	YES	YES	YES	YES
Year FE	YES	YES	YES	YES
Firm FE	YES	YES	YES	YES
City FE	YES	YES	YES	YES
Observations	1,915	1,915	1,171	1,172
Adj R^2^	0.592	0.634	0.379	0.447

**Note:** The annotation in this table remains consistent with that in Table 3.

### 4.2 Crowding out effect between cities

When faced with similar environmental constraints, there is often noticeable convergence in the decision-making of management regarding the same external information [77]. However, the demonstration effect of LCCP depends on the availability of various resources. The label signal of "pioneer" alleviates the pressure of resource competition in LCCP. This advantage will attract a large number of high-quality resources into pilot cities [78], as well as, it receives more resource supports from the central government. Therefore, firms in LCCP are getting more financial support, preferential policies and other environmental governance resources. In this way, "peer" firms (non-pilot areas in the same province) face increased competitive pressure and financing costs in obtaining environmental protection resources, leading to "crowding out effect".

**Fig. 4:**
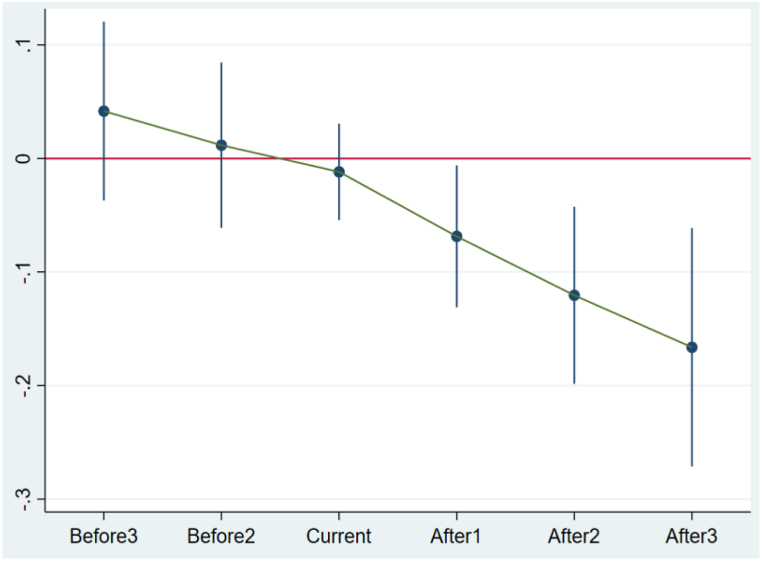
Parallel trend diagram of the crowding out effect model.

In order to provide additional insights into whether the LCCP policy has crowding-out effects on corporate ESG performance in surrounding cities, we initially remove the influence of the LCCP. Subsequently, we categorize the remaining cities within the province where the LCCP is situated into a treatment group, while the remaining cities serve as the control group. We consider the year 2013 and subsequent years as the implementation period. The results presented in Table 11 illustrate the influence of the LCCP policy on corporate ESG performance in neighboring cities over the ensuing four-year period. Regardless of *ESG*_*t+2*_*, ESG*_*t+3*_, or *ESG*_*t+4*_ as a dependent variable, the coefficients of Treat1*Post are all negative and significant. This result suggests significantly that LCCP has a crowd out effect on "peer" firms (different cities in the same province). Meanwhile, compared with LCCP, the impact in surrounding cities has a time lag, as well as passing the parallel trend test (see in Fig. 4).

**Table 11:** Crowding out effect between cities.

*Variable*s	(1)	(2)	(3)	(4)
	${ESG}_{t+1}$	${ESG}_{t+2}$	${ESG}_{t+3}$	${ESG}_{t+4}$
Treat1*Post	**−0.039**	−0.083**	−0.106***	−0.122***
	**(-1.06)**	**(-2.40)**	**(-3.57)**	**(-3.06)**
*Controls*	YES	YES	YES	YES
Year FE	YES	YES	YES	YES
Firm FE	YES	YES	YES	YES
City FE	YES	YES	YES	YES
Observations	13,095	13,095	13,095	13,094
Adj R^2^	0.689	0.698	0.675	0.680

**Note:** The annotation in this table remains consistent with that in Table 3.

### 4.3 Channel analyses

There are two main factors that motivate firms to enhance ESG performance. On the one hand, it roots in pressure from the external environment, such as the government, social supervision of public opinion and peer competitors. On the other hand, it stems from self-interested considerations, for example, demonstrating brand positioning, enhancing the firms' reputation, increasing market share and sales, and obtaining government support [79]. After the implementation of the policy, LCCP has a "supervision" function. The likelihood that companies will undergo a green transformation is increased the more the government pays attention to local environmental issues and the stronger the oversight of industry polluting operations [80]. Besides, local governments in the pilot areas will increase economic and administrative measures to guide firms to improve their future potential sustainable governance performance for the sake of their green political achievements [18].

Similarly, LCCP has the "publicity" function, which can guide the public privates, regarded as a part of stakeholder, to pay more attention to the environmental conditions in the region, and rise interest in and readiness to pay for environmental goods [81]. According to stakeholder theory, connected to corporate a legitimate claim, public privates affect corporate decision-making and behavioral performance and motivate companies to take environmental action [82]. As a social communication behavior, public attention exerts pressure on firms from the perspective of public opinion spread and urge them to shift corporate environmental protection behavior and improve their ESG performance [83].

To verify the potential channels, we analyze the mechanism from the perspectives of government environmental concerns, as well as, public environmental concerns. In the content of government concern for the environment (*Gov-Concern*), we utilize the ratio of environmental governance and green transformation terms documented in the local government work reports over the years in the total word count as the proxy variable. In the content of public concern for the environment (*Priv-Concern*), Baidu index for searching environmental pollution keywords in each city is used as a proxy variable. Table 12 of Columns (1) and (2) show the mechanism of government's attention to the environment. Columns (3) and (4) demonstrate the mechanism of public's attention to the environment and environmental awareness enhancement, forcing the firms to improve their ESG performance to meet the public's environmental demands.

**Table 12:** Mechanism analyses.

*Variable*s	(1)	(2)	(3)	(4)
	Gov-Concern	Gov-Concern	Priv-Concern	Priv-Concern
Treat*Post	0.061***	0.062***	3.329***	3.227***
	**(2.60)**	**(2.64)**	**(3.51)**	**(3.41)**
*Controls*	NO	YES	NO	YES
Year FE	YES	YES	YES	YES
Firm FE	YES	YES	YES	YES
City FE	YES	YES	YES	YES
Observations	9,513	9,513	8,619	8,619
Adj R^2^	0.428	0.428	0.904	0.904

**Note:** The annotation in this table remains consistent with that in Table 3.

## 5 Discussion and conclusions

With a series of serious pollution incidents have occurred all over the world, the necessity of corporate green transformation has been highlighted. This paper exploits the setting of LCCP and document that LCCP policy can significantly promote the corporate ESG performance in LCCP, especially in high-emission areas and high-emission industries. The channel analysis shows that, LCCP policy affects the local governments and the public's attention, forcing firms to actively engage in ESG practices. Furthermore, the LCCP policy exerts a crowding-out effect in surrounding areas. It implies that comprehensive environmental regulation represented by LCCP policy can help firms achieve green transformation, but attention should be paid to the synergy of surrounding areas.

Our study underscores the meaningful impact of LCCP policy on enhancing ESG performance. However, the efficacy of this policy is intricately linked to the carbon emission intensity in diverse regions and industries. It is noteworthy that unintended consequences arise, exemplified by the negative spillover effect of low-carbon cities on neighboring counterparts, leading to resource displacement. In light of these findings, policymakers face crucial considerations: (1) Central policy-makers must coordinate green development across regions, strategically planning and allocating resources to foster a pattern of "upward competition". (2) Regions designated as LCCP should leverage their role, particularly in areas with high carbon emissions, to maximize the incentivizing effects of low-carbon policies. (3) Surrounding areas not designated as low-carbon pilot cities must recognize potential resource displacement and actively encourage businesses to improve their ESG performance, aiming to secure more favorable policy support. In this holistic way, it ensures a balanced and effective implementation of low-carbon policies for sustainable development.

However, this paper has several limitations. Due to the LCCP policy is a comprehensive and bottom-up regional environmental regulation policy, there are certain differences in the intensity, ways and means of policy implementation in different regions. In this paper, we do not carry out a more detailed analysis and categorically discuss the different specific low carbon policies in certain pilot cites. In addition, ESG performance presents three conponents of corporates' sustainable development, that is, environment, social, and governance. Further studies can delve deeper into different dimensions of ESG to potential conclusions.

## Data availability statement

Data is available upon request.

## Funding statement

The authors would like to appreciate the financial support from the National Social Science Fund of China (grant number: 23BMZ065).

## CRediT authorship contribution statement

**Wenzhe Yu:** Writing – original draft, Software, Data curation. **Zhong Li:** Project administration, Methodology. **Caijuan Hu:** Funding acquisition, Conceptualization.

## Declaration of competing interest

The authors declare the following financial interests/personal relationships which may be considered as potential competing interests: Caijuan Hu reports financial support was provided by Chinese National Funding of Social Sciences. If there are other authors, they declare that they have no known competing financial interests or personal relationships that could have appeared to influence the work reported in this paper.

## Footnotes

1 On November 26, 2012, the second batch of LCCP established. According to An et al.(2016) [46] and Chen et al.(2022) [18], we set the first year affected by the policy shock to be 2013, and the Post = 1 in and after 2013.

## Footnotes

2 We also conducted a T-test for main variables between the city group and industry group. The result can be requested from the authors.

3 Data source: https://www.ceads.net.cn/.
